# First year experience with newly developed Leksell Gamma Knife^®^ Perfexion^™^

**DOI:** 10.4103/0971-6203.54848

**Published:** 2009

**Authors:** Jagdish P. Bhatnagar, Josef Novotny, Ajay Niranjan, Douglas Kondziolka1, John Flickinger, Dade Lunsford, M. Saiful Huq

**Affiliations:** Department of Radiation Oncology, Neurological Surgery, University of Pittsburgh Cancer Institute, Pittsburgh, USA

**Keywords:** Leksell Gamma Knife PERFEXION, Leksell Gamma Knife dosimetry, Leksell Gamma Knife treatment planning

## Abstract

A new model of Leksell Gamma Knife® (LGK), known as Perfexion™ (LGK PFX), was introduced by Elekta Instrument, AB, Sweden, in 2006. This model has a radically different design from the earlier models U, B, C and 4C. Dosimetric characteristics of LGK PFX, technical differences between LGK PFX and LGK 4C, experience gained with acceptance testing and commissioning of the LGK PFX, and comparison between LGK PFX and LGK 4C are presented in this study. Excellent agreement is found between the manufacturers recommended values of absorbed dose rate, relative output factors for 4 and 8 mm collimators, coincidence of mechanical and dosimetric isocenter, FWHM for beam profiles for various collimators and those reported in the present study. Excellent agreement is also found between the dosimetric characteristics of LGK PFX and LGK 4C for the 4 and 8 mm collimators. Examples of clinical cases treated with LGK PFX and impact of LGK PFX on workflow and dosimetric conformity of treatment planning is also given. The set up and treatment of patients on the LGK PFX is much more efficient since it is a fully automated system. The system also provides more options to generate plan with high dosimetric conformity.

## Introduction

Gamma Knife®, a device for stereotactic radiosurgery for the cranial lesions, was invented and basic principles of the stereotactic radiosurgery formulated by a Swedish neurosurgeon, Lars Leksell between 1950s and 1960s and finished by the installation of the first Gamma Knife® device in 1967.[1] Since then, this device has undergone several improvements with the advancement in technology. The latest version of this device, known as Leksell Gamma Knife PERFEXION™ (LGK PFX), (Elekta Instrument AB, Stockholm, Sweden) was introduced in 2006. The radiation unit of LGK PFX is re-designed with an entirely new beam geometry as compared to the previous gamma knife models U, B, C, and 4C.[2–11]

A LGK PFX system was installed at the Department of Neurological Surgery at the University of Pittsburgh Medical Center in August, 2007. A systematic approach was undertaken to perform acceptance testing, commissioning, and detailed measurement of various dosimetric characteristics of the system. The treatment unit has been in clinical use since September, 2007. The goals of this paper are manifold: i) present the experiences gained with the LGK PFX system during acceptance testing and commissioning; ii) report results of measurement of the dosimetric characteristics of the LGK PFX system; iii) present the results of the dosimetric comparison between LGK PFX and 4C; and iv) discuss experiences gained from use of the PFX system during the first year of clinical use.

## Materials and Methods

### Comparison of design characteristics of LGK PFX and previous LGK models

In LGK PFX, a total of 192 ^60^Co sources are arranged in a cylindrical configuration in five rings. This arrangement of sources differs substantially from the previous hemispherical arrangements of ^60^Co sources in the U, B, C and 4C units and results in different source-to-focus distance for each ring varying from 374 mm to 433 mm. The primary and secondary collimators of previous models have been replaced by a single large 120 mm thick tungsten collimator array ring. Consequently, there has been no requirement of collimator helmets for the LGK PFX system.

**Figure 1:**
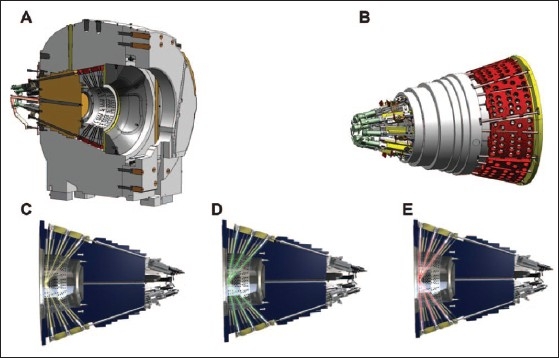
LGK PFX radiation unit and collimator system. A) Cross section of the LGK PFX radiation unit. B) Detailed view of sectors; each sector holds 24 ^60^Co sources and can be moved independent of other sectors in desired position to define a collimator size or to block beams. C) Sector position which defines a 4 mm collimator. D) Sector position which defines a 8 mm collimator. E) Sector position which defines a 16 mm collimator. (With permission of Elekta Instrument AB, Stockholm, Sweden.)

**Figure 2:**
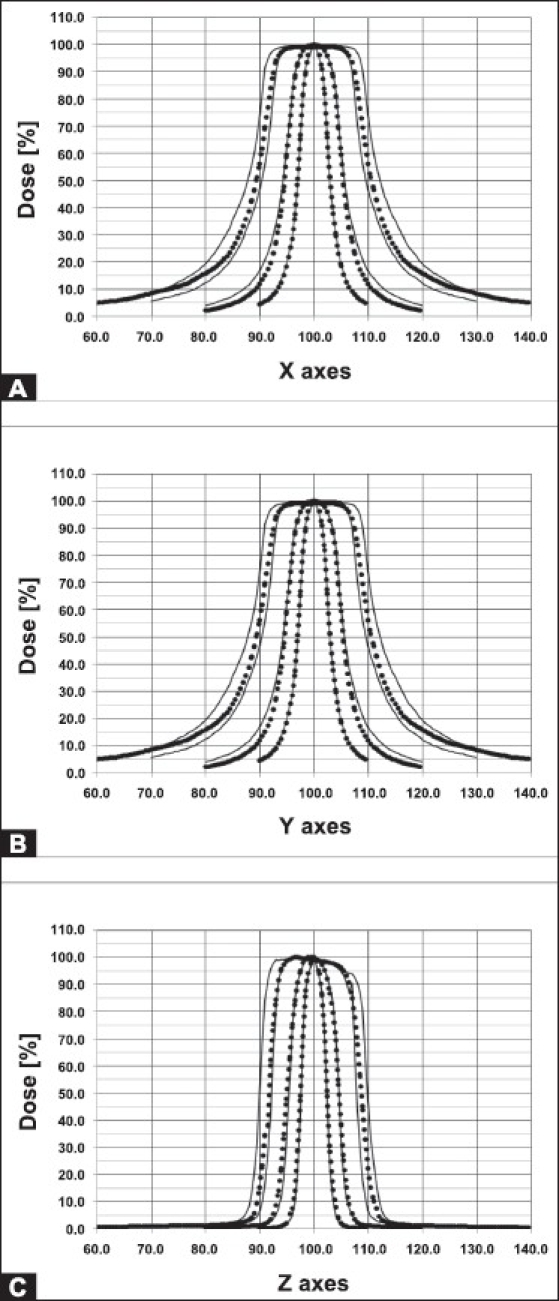
Comparison of LGK PFX and LGK 4C profiles calculated for 4, 8, 14, 16, and 18 mm collimators for all stereotactic X, Y, Z axes. Profiles for LGK 4C are given as solid lines whereas those for LGK PFX are given as dotted lines

For the LGK PFX system, the range of collimators (beam size) is changed from the previous gamma knife models. Only three collimator sizes of 4, 8 and 16 mm are now available. The 4 and 8 mm collimator sizes are kept the same as for the previous models, but the 14 and 18 mm collimator sizes of the previous models are replaced with a 16 mm collimator size. The 120 mm thick tungsten collimator ring is subdivided into eight identical regions, each region containing 72 collimators (24 collimators for 4 mm, 24 collimators for 8 mm, and 24 collimators for 16 mm). The beam size for each region is changed automatically by moving 24 sources over selected collimator set. A sector containing 24 sources can be moved into one of the five different positions: 1) sector moved to home position when the system is in a standby mode, 2) sector moved to 8 mm collimator size, 3) sector moved to 4 mm collimator size, 4) sector moved to 16 mm collimator size and 5) sector moved to off position which is the position between 4 mm and 8 mm collimators providing blocking of all 24 sources. The sector movement is performed by servo-controlled motors with linear scales located at the rear of the radiation unit. Detailed figure of the LGK PFX collimator system is given in Figure 1. Comparison of calculated profiles of LGK PFX and LGK 4C is given in Figure 2.

In the LGK PFX system, the range of couch movement around the focal point of radiation beams along the X, Y, and Z axes has been substantially increased. The mechanical treatment range in the X/Y/Z orientations is (160/180/220) mm for LGK PFX system as compared to (100/120/165) mm for other gamma knife models. Despite increasing the treatment range, the average source-to-focus distance is still kept very close to previous models due to an improved design of the collimator system of LGK PFX. This superior collimation was achieved by having only one collimator made of 120 mm thick tungsten ring, compared to a thicker collimating system of B, C and 4C models, which consist of primary and secondary collimators. Because of the increased treatment range for the LGK PFX system, the treatment of multiple brain tumors such as brain metastases does not present any difficulty in terms of collision with the collimator system.

In the LGK C and 4C systems, the stereotactic treatment coordinates are setup using the Automatic Positioning System (APS). This has been replaced by a patient positioning system (PPS) in the LGK PFX system. In PPS, instead of moving only the patient's head, the whole body of the patient lying on the PPS is moved into the pre-selected stereotactic coordinates. The PPS provides a more comfortable position for the patient during treatment. The majority of the treatments with LGK PFX can be completed in just one run with the gamma angle of 90°, i.e., patient's head in horizontal position. Occasionally, a change in gamma angle is required to avoid collision with the collimator cap which results in a second run for the treatment. The docking of the patient into the PPS is done by means of a frame adapter that attaches to the standard stereotactic Leksell G frame with the help of three clips. The adapter is then directly docked to the PPS. The patient's head can be locked in one of the three different gamma angles (head rotations): 70° (chin up position), 90° (horizontal position) or 110° (chin down position). The gamma angle is the only treatment parameter that requires manual set up. The PPS replaces the APS and the manual trunnion stereotactic coordinate set up. The reproducibility of the stereotactic coordinates set up in the LGK PFX system is better than 0.05 mm.

The radically new design of LGK PFX has had a great impact on the treatment planning software and the treatment planning. As each shot with LGK PFX consists of gamma ray beams coming from eight sectors and each sector has either 4, 8 or 16 mm collimation or complete beam blocking, there are now three possible approaches to the treatment planning: 1) use a shot/shots consisting of just one collimator size in all eight sectors, equivalent to the classical approach, as used in the earlier models of gamma knife; 2) use a composite shot/shots where any of the eight sectors can have 4, 8 or 16 mm collimator or even be completely blocked; 3) use dynamic shaping where an automatic procedure within the treatment planning system is used to block certain sectors to protect volumes defined as critical structures.

**Figure 3:**
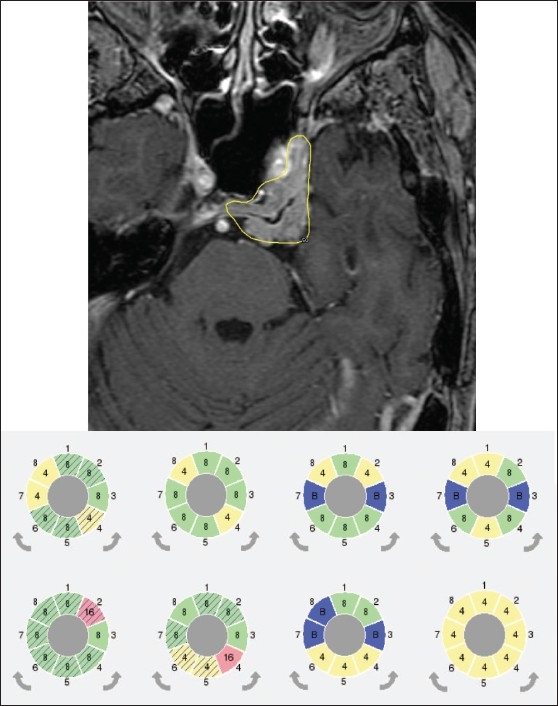
Example of using composite shots for the LGK PFX. To shape 50% isodose line and cover irregular target volume (meningioma) different combination of composite shots was used (see combination of eight sectors for each of eight different shots used). Different sector colors indicate different collimator size used: yellow – 4 mm, green – 8 mm, red – 16 mm and blue – blocked beams. Cross hatched sectors represent sectors that were automatically blocked by the treatment planning system to reduce dose to critical structures

**Figure 4:**
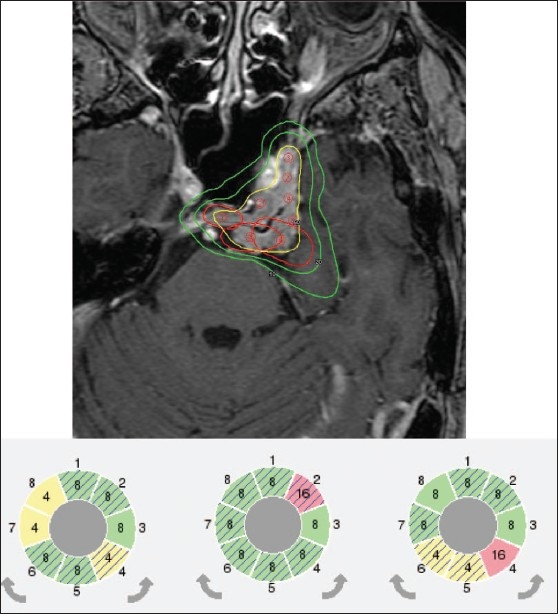
Example of using dynamic shaping for the LGK PFX. To shape 50% isodose line and cover irregular target volume (meningioma) and minimize dose to brainstem different sectors were blocked for three shots located close to brainstem by dynamic shaping. Please see elongated shape of these three shots and blocking pattern of sectors. Different sector colors indicate different collimator size used: yellow – 4 mm, green – 8 mm and red – 16 mm. Cross hatched sectors represent sectors that were automatically blocked by the treatment planning system to reduce dose to critical structures

The most significant change seen in treatment planning is the ability to generate a single isocenter composed of different beam diameters. Such a composite shot allows for optimized dose distribution shape for the individual shots.[12–15] Setup of any sector combination is almost free of time penalty during patient treatment since sector position changes are done automatically within five seconds. An example of using composite shots for the LGK PFX is given in Figure 3. Similarly, an example of using dynamic shaping for the LGK PFX is given in Figure 4.

The LGK PFX system also provides better patient and staff shielding.[12] Sectors are always in off position (blocked) during patient transportation in the treatment position, transition into new stereotactic coordinates, pause or emergency interruption. This results in significantly lower extra cranial radiation to the patient compared to the earlier models U, B, C and 4C.[12]

A comparison of various technical parameters between LGK PFX and LGK 4C is summarized in Table 1.

**Table 1:** Comparison of different technical parameters between LGK PFX and LGK 4C

*LGK PFX*	*LGK 4C*
192 Co-60 sources	201 Co-60 sources
Sources move	Sources stationary and fixed in position
Source-to-focus distance varies from 374 mm to 433 mm	Source-to-focus distance fixed at 400 mm
8 moving sectors holding 24 sources each	Sources fixed in central radiation unit
Collimators fixed - built in the unit	Final external collimators interchangeable
No collimator helmets	Four collimator helmets
Collimator sizes: 4, 8, 16 mm	Collimator sizes: 4, 8, 14, 18 mm
Automatic sector blocking	Individual manual beam blocking
Patient Positioning System (PPS) moves patient's whole body in X, Y, Z coordinates, requires patient frame adapter requires no patient frame adapter	Automatic Positioning System (APS) moves only patient's head in X, Y, Z coordinates'
No positioning tests required before treatment	Positioning tests required before treatment
No manual coordiantes set up	Manual coordinates set up possible
Treatment range in X/Y/Z (160/180/220) mm	Treatment range in X/Y/Z (100/120/165) mm
Gamma angles 70, 90 and 110 for patient's head fixation	Gamma angles 72, 90, 110, 125 (for APS) for patient's head fixation

Leksell Gamma, Knife PERFEXION™

### Acceptance, Commissioning and Quality Assurance for LGK PFX

This section gives a very brief description of a variety of tests which were conducted for the acceptance and commissioning of the unit. These tests are summarized in Table 2 together with tolerances as recommended by the manufacturer and adapted by the authors. Most of these tests also become part of routine daily, monthly or annual quality assurance tests.

**Table 2:** Acceptance Tests and Commissioning for the LGK PFX

*Item*	*Tolerance*
Acceptance Tests	
System initialization test	Functional
System normal treatment functionality test	Functional
Clearance test tool QA check	Functional
Safety and emergency tests	Functional
Uninterrupted power supply test	Functional
PPS radial precision test with central diode	50 μm for 4 mm collimator, 150 μm for 8 and 16 mm collimators
PPS radial precision test with two diodes in offset positions	300 μm for 4 mm
Radiological acceptance test (performed by ELEKTA):	
- dose rate measurement	Must be at least 3.000 Gy/min
- profiles	± 1.0 mm for FWHM
- coincidence of mechanical and dosimetric isocenter	0.4 mm
Commissioning Tests	
Absolute dose output at the center of 160 mm diameter polystyrene calibration phantom for 16 mm collimator	Must be at least 3.000 Gy/min ± 3.0%
Precision of beam alignment (measurement of X, Y, and Z dose profiles for 4, 8, and 16 mm collimators)	± 1.0 mm for FWHM
Coincidence of radiological focal point with PPS mechanical isocenter measured by film	0.4 mm
Measurement of Relative Output Factors (ROF)	± 2.0%
Measurement of sector output uniformity	± 1.0%
Timer-accuracy	± 0.1%
-linearity	± 2.0%
- constancy	± 0.1%
- end-effect	± 0.03 min
Sector transition dose	0.05 Gy for 16 mm collimator
End-to-end test including all steps of the treatment procedure	Functional
	Phantom delivered dose ± 4.0%
RPC MD Anderson SRS quality audit	Phantom delivered dose ± 5.0%
	Profiles offset ± 1.0 mm

Calibration of the LGK PFX was performed at the center of a 160 mm diameter ELEKTA polystyrene spherical calibration phantom using a miniature Exradin A16 (volume 0.007 cc) ion chamber connected to a Standard Imaging electrometer (Max 4000; Standard Imaging, Middleton, Wisconsin, USA). The recommendations of AAPM TG-21[16] protocol were followed for calibration.

Gafchromic EBT films (International Specialty Products, Wayne, New Jersey, USA) were used to measure dose profiles along the three stereotactic coordinates X, Y, Z for the 4, 8 and 16 mm collimators. Calibration doses used for EBT film ranged from 0 to 8 Gy (a total of ten calibration points were obtained). The films were read at least 24 hours after their exposure using a EPSON EXPRESSION 10000 XL scanner (Epson America, Inc., Long Beach, California, USA) with 200 dpi resolution in 48-bit color and imported in red channel. Scanning of the films was performed using the same EPSON scanner described above and analysis of data were performed using Film QA version 2.0.1215 software (International Specialty Products, Wayne, New Jersey, USA). Background corrections were applied to all films. The Full-Width-at-Half-Maximum (FWHM) were obtained from measured beam profiles and compared with manfacture values stored in the treatment planning system.

The same films, scanner and software were also used to measure relative output factors (ROFs) for the 4 and 8 mm collimators. Measurements of ROFs were repeated five times for each of collimator. The plus/minus 2.0 % tolerance limit for the measurement of Relative Output Factors (ROF) is taken from AAPM TG-40, Table 1.[19] The TG-40 recommendation is a general recommendation for field size dependence for output constancy of ^60^Co teletherapy units.

Coincidence of mechanical and dosimetric isocenter was evaluated by two diferent methods. In the first method, a specially designed Diode Test Tool manufactured by ELEKTA and delivered together with LGK PFX system was used for measurements. This diode detector can scan X, Y, Z profiles for 4, 8 or 16 mm collimators and dosimetric center for each axis can be calculated from the three measured profiles. Results of these measurements can then be compared with reference values stored in the system. In addition to the diode measurements, film method was also used. Coincidence of mechanical and dosimetric isocenter was established by using a special phantom which has a small needle located exactly at the geometric isocenter and can hold EBT films in the central position. Before exposing the films to radiation, the films were punched with a needle. The created pin prick was then compared with the center of the profile obtained from film analysis.

The measurement of sector output uniformity is also recommended for commissioning for a variety of reasons; (1) any of the 24 sources on each of the eight sectors may be missing or misaligned, or may have a different activity, (2) the speed of motors for moving the sectors in position for irradiation may be different with a different transit dose added at isocenter, (3) the collimator holes in some sections of tungsten ring may get misaligned during transportation and installation. Non-uniform output from the sectors will affect the isodose pattern during irradiation but such an effect will not be accounted for by the Leksell GammaPlan software, which assumes output from each sector to be uniform.

## Results and Discussion

During commissioning of the LGK PFX system the absorbed dose rate was measured using the 16 mm collimator. At the center of 160 mm diameter ABS phantom (polystyrene equivalent) provided by ELEKTA, this dose rate was found to be 3.633 Gy/min. The manufacturer's specification required initial output to be more than 3.000 Gy/min. For almost the same initial activity as for the LGK PFX, the initial output measured for the LGK 4C and 18 mm collimator was 3.577 Gy/min. In spite of the fact that LGK PFX radiation cavity is 300% larger, the output measured for LGK PFX is still comparable to that measured for LGK 4C since source to focus distance is almost the same for both units. A slightly higher output of LGK PFX is probably related to the improved design of the collimators in LGK PFX.

Results of measured relative output factors (ROFs) for the 4 and 8mm collimators are presented in Table 3. Excellent agreement (to within 0.8%) is observed between manufacture recommended values of ROFs and present measured values.

**Table 3:** Results of relative output factor (ROF) measurements with EBT film dosimetry and their comparison with Monte Carlo (MC) calculations done by ELEKTA INSTRUMENT AB, Stockholm, Sweden for LGK PFX. Deviations between measured and calculated values are given in parenthesis. Relative output factor for 16 mm collimator size is by definition 1.000

*Collimator size*	*MC calculated ROF*	*Measured ROF by EBT film dosimetry*
8 mm	0.924	0.917 ± 0.014 (−0.8%)
4 mm	0.805	0.810 ± 0.007 (+0.6%)

Results for measured X, Y, Z profiles for 4, 8 and 16 mm collimators are given in Table 4. The measured FWHMs were found to be within 0.5 mm of the FWHM values quoted by the manufacturer. The manufacturer's specification required agreement within 1.0 mm. Figure 2 shows a comparison of profiles for all collimators between the LGK PFX and LGK 4C models. Although the design characeristics of LGK PFX are significantly different from those of the previous models, an excellent agreement is seen among 4 mm and 8 mm collimators profiles.[17]

**Table 4:** Results of FWHM measurements with EBT film dosimetry for LGK PFX

*Collimator size*	*Leksell Gamma Knife Perfexion FWHM*					
	
	*Expected FWHM [mm]*			*Measured FWHM [mm]*		
		
	*X*	*Y*	*Z*	*X*	*Y*	*Z*
4 mm	6.0	6.0	5.0	6.4	6.3	5.2
8 mm	10.7	10.7	9.7	11.0	10.9	9.9
16 mm	21.2	21.3	17.1	21.7	21.7	17.4

Accuracy of coincidence of radiological focal point and PPS mechanical isocenter was verified for the 4 mm collimator using the film technique described in the Materials and Methods section. The measured deviations by film method in the stereotactic coordinates were found to be as follows: ΔX = 0.00 mm, ΔY = 0.05 mm and ΔZ = 0.26 mm. The total radial deviation, calculated as square root of the sum of squares of individual ΔX, ΔY and ΔZ deviations, was 0.26 mm. This result was much better than manufacture required specification 0.4 mm.

The diode test tool yielded results for radial deviation of the central diode position that were well within the manufacturers recommended values. For the 4, 8 and 16 mm colimators, these deviations were found to be 5 *μ*m (specification 50 *μ*m), 41 *μ*m (specification 150 *μ*m) and 97 *μ*m (specification 150 *μ*m) respectively. Additionally, radial deviation for two offset diode positions for the 4 mm collimator were found to be 16 *μ*m and 43 *μ*m (specification 300 *μ*m).

All system functionality and safety tests were according to the specifications required by the manufacturer.

Timer accuracy, linearity, constancy and end-effect were within specifications as given in Table 2. Sector transition dose was measured to be 0.035 Gy for 16 mm collimator.

### Clinical experience with LGK PFX and comparison with previous systems

**Figure 5:**
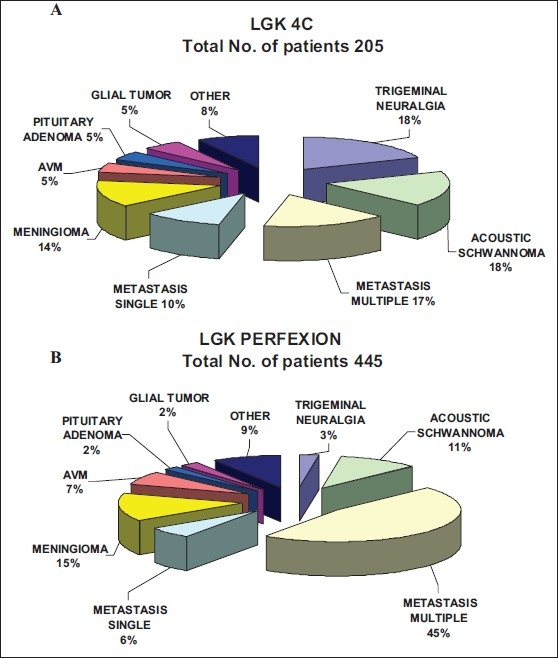
Comparison of patient distribution according to diagnosis between (A) LGK 4C and (B) LGK PFX treated during the first year after LGK PFX installation at the University of Pittsburgh Medical Center

Both LGK 4C and LGK PFX are clinically active systems used for patients' treatments at the University of Pittsburgh Medical Center. During the first year of clinical use, 445 patients were treated on LGK PFX. During the same period, the number of patients treated on LGK 4C was 205. Figure 5 shows the distribution of patients treated during this time on the LGK PFX and 4C according to their diagnosis. After the installation of LGK PFX, we noticed a remarkable change in the pattern of patients treated on LGK PFX and LGK 4C. Due to the convenience of automated set up and efficiency of treatment without physically changing collimators and using trunnions, LGK PFX became the unit of choice for the treatment of multiple metastases or targets with complex shape or larger volume. For the same reason, the number of patients scheduled for treatment on LGK PFX more than doubled the number of patients scheduled for treatment on the 4C unit.

To quantify treatment efficiency and dosimetric conformity of LGK PFX and LGK 4C, almost identical treatment planning was carried out for first 37 patients (21 with multiple brain metastases and 16 with benign tumors) for both LGK PFX and LGK 4C using the Leksell GammaPlan Version 8.0 TPS. Iso-dose plans were generated for the same doses as were prescribed for treatment. Total treatment times and conformity indices were compared for both LGK 4C and LGK PFX generated treatment plans. A brief discussion on conformity index (CI), its modification by Paddick (PCI)[20] and a complementary quantity to CI known as gradient index (GI) as proposed by Paddick and Lippitz[21] is given in Appendix-A.

Twenty-one patients underwent radiosurgical treatment of multiple brain metastases using 3-21 (median 9) isocenters on LGK PFX. These patients would have been treated using three-22 shots (median 8) on LGK 4C. No significant difference was observed between LGK 4C and LGK PFX for total beam-on time in the case of multiple metastases. However, the median reduction in set-up time for treatment of multiple metastases on the LGK PFX was 53 min per patient (range 19-125 min) which was statistically significant (p less than 0.01, Student's t-Test). The time saved for set-up was proportional to the number of irradiated tumors (maximum time saved for set-up was 125 min for a patient with 12 metastases).

All benign tumor radiosurgery treatments were performed in a single run comprising of a median of 13 isocenters (range 5-24) on LGK PFX. The median reduction in set-up time for treatment of a benign tumor on the LGK PFX was 16 min per patient (range 5-53 min) which was statistically significant (p less than 0.01, Student's t-Test).

The median values for Conformality Index (CI), Paddick Conformality Index (PCI) and Gradient Index (GI) (exact definition of all three conformality indeces can be found in[20–21] for LGK PFX were 1.11, 0.81 and 2.67 respectively and that for LGK 4C were 1.08, 0.86, and 2.78 respectively. No significant difference was seen in the conformity using CI, PCI and GI indices between LGK 4C and LGK PFX treatments. Details on comparison of efficiency and conformality between LGK PFX and LGK 4C has been given by Niranjan *et al*.[18]

There were no major technical issues related to the LGK PFX observed during our first year experience. Clinical down time when the system could not be used for the patients' treatment was zero. Due to wider mechanical treatment range in the X/Y/Z coordinates, the treatment for all patients with multiple brain metastases could be performed in one single session instead of dividing the whole treatment in two or three individual treatment sessions with different stereotactic frame placement. Total treatment time on the LGK PFX even for patients with very complex treatments (10-15 brain tumors) never exceeded two hours.

## Conclusions

The first year experience with the LGK PFX at the University of Pittsburgh Medical Center demonstrated high reliability and consistency in accuracy and functionality of the system. Daily, monthly and annual quality assurance tests were always within the specifications. The LGK PFX demonstrated a high level efficiency in workflow thus reducing significantly the total treatment time especially for the treatment of multiple metastases. The new design features of the system offer many dosimetric capabilities to create highly conformal treatment plans. The unit has worked remarkable well with zero down time.

## Appendix A

***Conformity index (CI), paddick conformity index (PCI) and gradient index (GI)***

The indices discussed in this appendix are useful tools to compare competing treatment plans for the same patient.

The concept of giving the prescribed dose to the target volume (TV) and reducing the dose to the surrounding healthy tissue is objective measure by the quantities known as CI, PCI and GI. The CI was originally proposed in 1993 by the Radiation Therapy Oncology Group radiosurgery guidelines.[22] The CI is expressed as,

(1) CI=PIV/TV

where PIV is the “prescribed iso-dose volume”.

Paddick suggested that the definition of CI as given in Eqn. I assumes that both the PIV and the TV have same isocenters and that both are of same shape. In certain circumstances, when iscocenters of PIV and TV are different and so are the shapes, it is possible to calculate CI as having a perfect score of “1” without this actually being the case. In order to remove this flaw, Paddick suggested an alternate quantity, known as PCI, with the following expression,

(2) $$\mathrm{PCI}={\mathrm{TV}}_{{\mathrm{PIV}}^{2}}/(\mathrm{TV}\times \mathrm{PIV})$$

where TV_PIV_ stands for the target volume which overlaps with PIV.

With this definition, the false perfect scoring of CI is eliminated.

The gradient index (GI) provides a measure of spread of low dose outside TV into the surrounding healthy tissue. GI is calculated by,

(3) $$\mathrm{GI}=({\mathrm{PIV}}_{50\%})/(\mathrm{PIV})$$

where PIV_50%_ is the volume of the half the prescription iso-dose.

Keeping a high score of PCI indicates good conformity of a treatment plan and keeping a low value of GI indicates low dose to the surrounding tissue.
